# Correspondence: Iron deposition in steatotic liver disease—A matter of compartment, but can it be a target for treatment?

**DOI:** 10.1097/HC9.0000000000000995

**Published:** 2026-07-15

**Authors:** Noel Ravindranayagam, Karl Vaz, Monique Fernandez, Julie Lokan, Andrew Grigg, Marie Sinclair

**Affiliations:** 1 Department of Surgery, Victorian Liver Transplant Unit, Austin Health, Heidelberg, Victoria, Australia; 2 Department of Medicine, University of Melbourne, Parkville, Victoria, Australia; 3 Department of Anatomical Pathology, Austin Health, Heidelberg, Victoria, Australia; 4 Department of Haematology, Austin Health, Heidelberg, Victoria, Australia

To the editor,

We read with interest “Association between pattern of hepatic iron deposition and severity of MASH among patients enrolled in the NASH Clinical Research Network” by Sohal et al,^1^ which explored the compartment of hepatic iron deposition on metabolic dysfunction–associated steatohepatitis (MASH) histologic outcomes. The authors analyzed liver biopsies from 2833 participants in the NASH Clinical Research Network and 2 large MASH pharmacotherapeutic randomized controlled trials (RCTs), of which 41% (1166) had positive hepatic iron staining and included people across the spectrum of fibrosis (no fibrosis to cirrhosis). In stratifying participants according to the compartment of hepatic iron deposition [hepatocellular, reticuloendothelial system (RES) or mixed], the authors determined RES iron staining to be associated with a greater NAFLD Activity Score (NAS) and increased fibrosis stage than those confined to the hepatocellular or mixed compartments, and those with no evident hepatic iron staining. Furthermore, RES hepatic iron deposition was an independent predictor of fibrosis after adjustment for age, sex, diabetes, and body mass index. We commend the authors in harnessing the power of large cohorts to allow detection of small differences between groups to be established.

Given the mixed literature on the influence of raised iron indices in metabolic dysfunction–associated steatotic liver disease (MASLD), we performed a single-center retrospective analysis on 127 participants with biopsy-proven MASLD between 2010 and 2019 to investigate the relationship between hyperferritinemia, iron deposition on liver histology and liver fibrosis. A smaller proportion (25/127, 20%) had histologic evidence of iron deposition in our study, while 30% (38/127) had hyperferritinemia (>300 μg/L for men, >200 μg/L for women). In those with hepatic iron deposition, 36% (n=9) each had isolated hepatocellular or isolated RES staining, while 28% (n=7) had mixed compartment staining (all grades 1–2 for hepatocellular staining, only 2 with more than mild RES staining).

Akin to the study by Sohal et al,^1^ those with hepatic iron deposition had significantly higher serum ferritin levels (255 μg/L vs. 113 μg/L, *p*=0.04) and a greater proportion had hyperferritinemia (52% vs. 25%, *p*=0.01) compared with those without hepatic iron deposition. We found no difference in rates of advanced fibrosis in those with and without hepatic iron deposition (32% vs. 31%, *p*=0.94), no correlation between serum ferritin and any grade of histologic fibrosis, either as a continuous variable for ferritin (Spearman rho = 0.118, *p*=0.18) or a categorical variable for hyperferritinemia (Spearman rho = 0.153, *p*=0.09), nor any difference in proportion of advanced fibrosis in those with and without hyperferritinemia (32% vs. 31%, *p*=0.94). Our study is likely to been underpowered to detect such differences and notably given the small numbers, further analysis through subcategorizing participants according to compartment of iron overload would have further impacted the statistical power.

We demonstrated hyperferritinemia [odds ratio (OR) 3.12, 95% confidence interval (CI) 1.22–7.96] to be an independent predictor for hepatic iron deposition after adjustment for age, gender, body mass index, diabetes, dyslipidaemia, and hypertension. However, only increasing age (OR 1.08, 95% CI 1.03–1.12) and prevalent diabetes (OR 3.86, 95% CI 1.42–10.53) were independent predictors for advanced fibrosis/cirrhosis after the same multivariate logistic regression model.

While larger cross-sectional studies have demonstrated an association between hyperferritinemia or histologic hepatic iron deposition and deleterious clinical and histologic outcomes,^1^^,^^2^ the existing therapeutic data utilizing venesection for iron-depletion remains mixed.^3^ In the largest RCTs assessing the role of therapeutic venesection in those with MASLD (n=74) or those with dysmetabolic iron overload syndrome (DIOS, n=274), there was no difference in markers of insulin sensitivity/resistance, serum transaminases nor hepatic steatosis despite a reduction in serum ferritin in those with MASLD,^4^ with no improvement in Fibrosis-4 Index or serum transaminases in those with DIOS.^5^ Aggressive venesection may also be poorly tolerated and was associated with weight gain in the French RCT.^5^ A smaller paired-biopsy RCT (n=21) over 24 months demonstrated an improvement in NAS without worsening fibrosis in those who undertook regular venesection compared with standard diet/lifestyle advice alone.^6^

Therefore, future studies are required to determine if RES compartment hepatic iron deposition can be a target for intervention through iron-depletion therapy (venesection, chelation, or hepcidin-mimetics) in people with MASLD, and if there are accurate and viable non-invasive tests to pragmatically select people for such treatment given the issues pertaining to serial liver biopsy, including patient acceptance. In any case, the current study and our own analysis suggest only a very small proportion of those with MASLD (<10%) would be eligible for such a therapeutic trial.

## References

[1] Sohal A Wilson LA Kleiner DE Loomba R Yuan L Dasarathy S . Association between pattern of hepatic iron deposition and severity of MASH among patients enrolled in the NASH Clinical Research Network. Hepatol Commun. 2025;9:e0847. 10.1097/HC9.0000000000000847 PMC12637332 41264906

[2] Armandi A Sanavia T Younes R Caviglia GP Rosso C Govaere O . Serum ferritin levels can predict long-term outcomes in patients with metabolic dysfunction-associated steatotic liver disease. Gut. 2024;73:825–834. 10.1136/gutjnl-2023-330815 38199805

[3] Fernandez M Lokan J Leung C Grigg A . A critical evaluation of the role of iron overload in fatty liver disease. J Gastroenterol Hepatol. 2022;37:1873–1883. 10.1111/jgh.15971 35906772

[4] Adams LA Crawford DH Stuart K House MJ St Pierre TG Webb M . The impact of phlebotomy in nonalcoholic fatty liver disease: A prospective, randomized, controlled trial. Hepatology. 2015;61:1555–1564. 10.1002/hep.27662 25524401

[5] Laine F Ruivard M Loustaud-Ratti V Bonnet F Cales P Bardou-Jacquet E . Metabolic and hepatic effects of bloodletting in dysmetabolic iron overload syndrome: A randomized controlled study in 274 patients. Hepatology. 2017;65:465–474. 10.1002/hep.28856 27685251

[6] Valenti L Fracanzani AL Dongiovanni P Rovida S Rametta R Fatta E . A randomized trial of iron depletion in patients with nonalcoholic fatty liver disease and hyperferritinemia. World J Gastroenterol. 2014;20:3002–3010. 10.3748/wjg.v20.i11.3002 PMC3961977 24659891

